# Six years of spreading BLSD skills in schools: empowering teachers as trainers

**DOI:** 10.3389/fpubh.2024.1454603

**Published:** 2024-10-15

**Authors:** Sara Calicchia, Silvia Capanna, Mariangela De Rosa, Bruno Papaleo, Alessandra Pera

**Affiliations:** Health Surveillance and Health Promotion Laboratory, Department of Occupational and Environmental Medicine, Epidemiology and Hygiene, Italian Workers' Compensation Authority (INAIL), Rome, Italy

**Keywords:** Basic Life Support and Defibrillation (BLSD), cardiopulmonary resuscitation (CPR), teachers, students, school, education

## Abstract

**Introduction:**

To increase the population's ability to handle emergencies, life-saving maneuvers should be universally acquired as an automatic skill starting from school through frequent and practical retraining. Teachers could be ideal multipliers, but it is necessary to design pathways that train and motivate them, since Basic Life Support and Defibrillation (BLSD) skills are not part of their academic curricula. This study explores innovative training solutions and facilitating factors to enhance BLSD education in schools by focusing on the training and motivation of teachers.

**Methods:**

In the 1st year, an in-service training program for teachers was provided and assessed in a lower middle school in central Italy. The study compared the skills of a group of students trained by teachers with those trained by certified instructors, immediately after the course and 6 months later. Both the procedural skills and the quality of cardiopulmonary resuscitation (CPR) were evaluated, through a hands-on simulation. Over the next 5 years, the project was expanded to three more schools, and a field study was conducted where researchers monitored the teachers in their classroom work, supporting them in resolving critical issues.

**Results:**

Quantitative assessments showed significant differences in student abilities immediately after the course, which diminished after 6 months. Qualitative evaluations indicated that teachers, initially reluctant, gained confidence and effectively taught BLSD skills. Over 5 academic years, the project expanded to additional schools, training 5,661 students in life-saving maneuvers with a ratio of 1 teacher for every 111 trained students, over the whole period of time. Sustainability factors included regular retraining of teachers, continually updated and free didactic materials and equipment, internal coordinators, allowing teachers the freedom to structure a customized course schedule in terms of timing and delivery methods.

**Conclusion:**

The study highlights the effectiveness of trained teachers as BLSD knowledge multipliers. Standardizing teacher training, including follow-up retraining, is crucial, but flexibility in student training is beneficial, allowing adaptation to the specific needs of schools. Future research should focus on the long-term sustainability of chain training in larger areas, identifying strategies to overcome organizational and motivational barriers.

## Introduction

Bystander cardiopulmonary resuscitation (CPR) is a crucial factor for the survival of individuals experiencing out-of-hospital cardiac arrest (OHCA) (1, 2). Data from these studies indicate that patients receiving bystander CPR exhibit higher rates of return of spontaneous circulation (ROSC) and survival to hospital discharge compared to those treated solely by emergency medical services (EMS). Specifically, EuReCa TWO reported an average bystander CPR rate of 58%, with notable variations between countries. The study revealed that 33% of patients achieved ROSC, and 8% survived to hospital discharge. According to the EuReCa TWO in Italy, the bystander intervention rate (32.7%) is below the European average. A low mean is also confirmed by a recent literature review, which found that bystanders initiated CPR in only 26% of OHCAs and used an automated external defibrillator (AED) in just 3.2% of cases (3).

One major barrier preventing ordinary people from intervening in emergencies is the fear of making mistakes, but bystanders who have received CPR training are more likely to intervene (4). Providing CPR training to laypeople is an integral part of an overall strategy to improve the population's propensity to intervene (5–8).

Among the various settings for disseminating CPR techniques, schools are currently a priority. Teaching students has proven effective for the acquisition and retention of knowledge and practical skills, and it enables ongoing resuscitation training (9–11). This could change the attitude and behavior of young people at a time in their lives when new information is easily absorbed (12, 13) and inhibitory barriers are lower (14). Additionally, schools provide excellent access to a large part of the community, including household members, and help to overcome social class barriers (15–18).

In line with scientific evidence, policies are also moving in this direction. In Europe, the “Kids Save Lives” statement, endorsed by the World Health Organization (WHO), has been a driving force behind the dissemination of school programs and awareness-raising campaigns for children and general population (19, 20). However, simply recommending or mandating the inclusion of CPR training in school curricula, although essential, does not always achieve the desired impact (5, 21), especially when there is poor coordination between schools and policies (16). Therefore, everything depends on the willingness and interest of individual schools and local communities (school administrators, students, and parents) (22, 23). This issue, combined with insufficient financial support, absence of systematic organization, and lack of standardization in training (16), makes it challenging to reach a critical mass of students and to ensure them adequate retraining and retention of CPR skills (24, 25). Other challenges include the continuous updating of teaching methods: flipped classroom models, realistic simulations using virtual and augmented reality, smartphone apps, and serious games have been shown to enhance Basic Life Support and Defibrillation (BLSD) skills and increase student engagement (10, 26).

Integrating BLSD training into the teachers' training curricula is a key factor in overcoming these barriers and addressing new challenges, first and foremost the need to ensure frequent retraining (10, 24). Training courses conducted by appropriately trained teachers have proven as effective as those conducted by healthcare professionals (17, 27). Additionally, teachers play a key role as facilitators and multipliers of skills (27). Potentially, teachers agree on the importance of including BLSD in school curricula, but not all feel capable or motivated to teach it. A survey conducted in Spain on a sample of 3,423 teachers reported that 98% of respondents support the KIDS SAVE LIVES declaration and agree with the inclusion of first aid training in schools and university degree programs (28). The main barriers to motivation include a lack of institutional support and gaps in BLSD knowledge. A recent study conducted in Belgium, where BLS became part of the secondary school curriculum in September 2010, highlighted that only 36% of teachers were aware of this, and most of those who did not feel capable of providing training had not attended even a basic training course (22). Age and perceived level of knowledge significantly impact teachers' motivation (16). Similar evidence has been reported in a German study, which also mentioned the absence of BLSD among school educational objectives and time constraints as barriers (29). Another barrier is the inconsistency in training paths: courses generally differ in duration, structure and materials used (16).

Italy also joined the KIDS SAVE LIVES initiative with a Legislative Decree that recommended teaching first aid in schools, along with ministerial guidelines suggesting educational objectives and training content for each age group. Despite this, there is no coordination between the political level and educational institutions. The most structured project, promoted by the Ministry of Education, was delivered to students through the 118 emergency services, while other initiatives targeting teachers remain confined to the local level (30).

In summary, recent studies have highlighted the effectiveness of teacher-led training and the factors that facilitate or hinder the implementation of resuscitation training programs for schoolchildren. The next step involves exploring innovative solutions through field experience that closely aligns with the needs of schools and their teachers, ensuring that their voices are heard and addressed (31).

The present 5-year study aimed to evaluate the effectiveness and long-term sustainability of a train-the-trainer BLSD program designed for lower middle school teachers. Initially, the effectiveness of the training pathway was evaluated in one school through a comparison of the results of students trained by qualified instructors and those taught by appropriately trained teachers. The validated pathway was then replicated in three additional schools and monitored over 5 years to evaluate long-term sustainability.

## Methods

**Figure 1:**
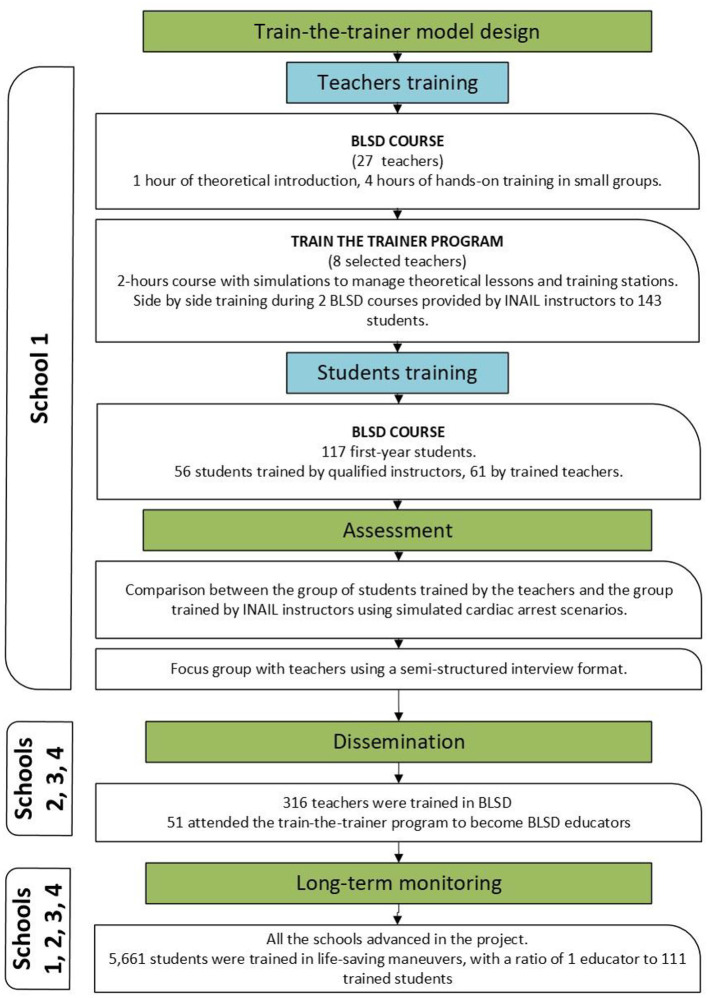
Study design.

The so called project “A scuola di RCP!Rianimare Ci Piace! [tn: We like to study and perform resuscitation]” was conducted by the National Institute for Insurance against Accidents at Work (INAIL), accredited as a BLSD Training Center by Emergency Operations Center (Ares 118 Lazio). The members of the research team were BLSD instructors: two physicians and six laypeople, trained to teach lay people according to the standard provided by the European Resuscitation Council (ERC) Guidelines (32) and certified by the Italian Resuscitation Council for the Community (IRC Comunità), The idea was developed within a Public Access Defibrillation (PAD) project implemented in the municipality of Monteporzio Catone (RM) and later expanded to additional three schools. The research project was approved by the Research Department's management of INAIL and the schools formally requested to join. All courses were scheduled during school hours, and the teachers informed their students about the program. The study can be summarized in the following phases (Figure 1).

### Design of the training model for teachers

The in-service training program for teachers was designed based on the train-to-trainer program usually adopted by IRC Comunità, an association that has been delivering specific training courses for laypeople throughout Italy.

### Teachers and students training

Twenty-seven teachers from a lower middle school (students' age 11–14) in central Italy (Don Lorenzo Milani, Monte Porzio Catone) were involved in a BLSD course during the 2017–2018 academic year. All the schoolteachers received the same instruction, as follows (Table 1): 1 h of theoretical introduction and 4 h of hands-on training in small groups with a 1/6 instructor/participants ratio, according to the standards provided by the ERC Guidelines (32). The didactic content of the basic BLSD module also reflects the ministerial guidelines on First Aid in schools, except for the treatment of trauma and burns.

**Table 1:** Standard BLSD responder course (5 h).

**Step**	**Specifications**	**Tools**
Theoretical lesson (1 h)	In plenary	Slides, video
Practical training on BLSD (3 h) - Environment, consciousness, and breath assessment. - CPR (insufflation only before COVID-19) Use of AED	The entire BLSD sequence was taught in training stations consisting of a maximum of 6 learners per 1 instructor, according to the following scheme: **Phase 1:** the instructor demonstrates the maneuvers on the manikin without commenting. **Phase 2:** the instructor comments on the maneuvers while repeating them on the manikin. **Phase 3:** the learners instruct the instructor on how to perform the maneuvers. The instructor executes without anticipating. **Phase 4:** the learners repeat the maneuvers in turn aided by the Laerdal QCPR app. **Phase 5:** structured feedback from the instructor	Laerdal Little Anne QCPR manikins + Laerdal QCPR app + AED trainer
Practical training: infant, child, and adult choking maneuvers (30 min)	The choking maneuvers are explained by the instructor and repeated in turn: on the manikin in the case of the infant chocking, among peers in the case of adult choking	Little Baby manikin
Practical training: recovery position (20 min)	Paired simulation.	
Check out (10 min)		Summary posters with choking and BLSD maneuvers

Based on their skills and motivation, eight teachers were selected to become BLSD educators by INAIL staff, in agreement with the head of the school. Teachers attended an additional 2-h course designed to transfer the main techniques to manage both the theoretical lesson and the training station. Then, they were engaged in a “side-by-side” training during two BLSD courses provided by INAIL instructors to 143 3rd-year students (ages 13–14): in the first course, the teachers only assisted the INAIL personnel; in the second one, they managed the training stations under the instructor's supervision. At the end of the course, feedback on strengths and areas for improvement was provided to the teachers.

Subsequently a convenience sample of 117 1st-year students (age 11–12), none of whom had previous experience, attended to a BLSD course (Table 1): 56 students were trained by qualified instructors, and 61 by trained teachers, maintaining a 1/6 instructor/participants ratio. Students were engaged by the teachers, who explained the research objectives to them.

### Quantitative and qualitative assessment

**Figure 2:**
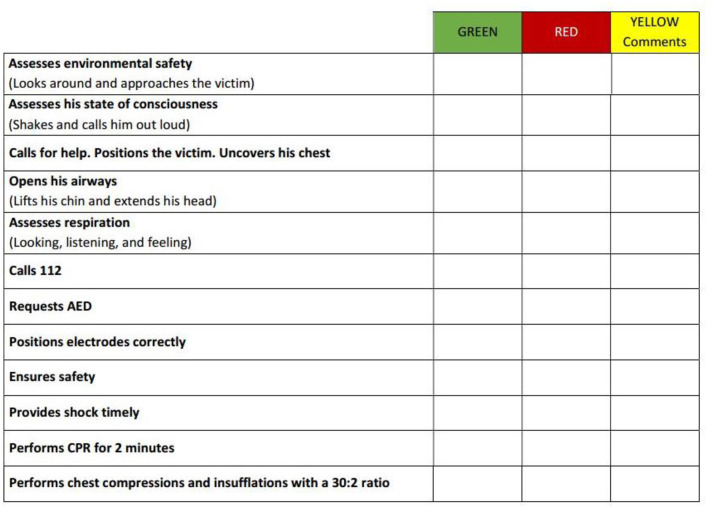
Skill test for the evaluation of the BLS/AED sequence.

The abilities of the two groups were assessed immediately after the course and again 6 months later using a simulated cardiac arrest scenario. The assessment was conducted by three of the instructors who were present during the initial training phase: one physician and two lay instructors (non-healthcare personnel). They had previously agreed on what to communicate to the students and how to manage the scenario. At the beginning of each assessment session, the research objectives and tasks were explained to the students, who then gave verbal consent to proceed. For the 6-month assessment, to prevent information spread and suggestions, the students were not informed of the scheduled test and were called separately. Students were told that they had to imagine someone close to them falling to the ground. A manikin placed on the floor played the role of the victim and they were required to act. Both the procedural skills and the quality of CPR were evaluated. The procedural skills was assessed through a skill test (Figure 2): researchers assigned a green mark for correct steps, red for completely wrong or omitted steps, and yellow for partially correct or not perfectly sequenced steps. The quality of CPR was assessed through a hands-on simulation using a QCPR training manikin with the Laerdal PC SkillReporting System, which recorded:

- Chest compressions score (depth, rate, release, hand position, and frequency).
- Flow fraction score (percentage of the time compressions were given).
- Ventilation score (volume and frequency).
- Total score (overall score calculated from compression, flow fraction, and ventilation scores).

At the end of the school year, the teachers participated in a focus group conducted by two members of the research team with previous experience in qualitative research. Using a semi-structured interview format (33), the discussion covered the following topics: suggestions for the project, effectiveness of the training course in transferring knowledge to students and any difficulties encountered in teaching, scheduling and organizational procedures.

### Analysis

For the purpose of quantitative analysis, during the correction of the skill test, 0 was assigned for a wrong answer, 1 for a partially correct answer, and 2 for a correct answer; the scores of each part of the sequence were added up to a maximum of 24 points. Statistical analysis conducted with the software SPSS^®^ 25.0 (IBM^®^ Corporation, New York, NY, USA) compared the results obtained by the group of students trained by the teachers to those of the group trained by the INAIL instructors. The Kolmogorov-Smirnov test was used with a 0.05 *p*-value to study data distribution. For data with a normal distribution, differences between the two groups (teachers-INAIL instructors) were calculated with the *T*-test, while for non-normally distributed data, the non-parametric Mann-Whitney test was used, both with a *p*-value of 0.05. For normality verification, and subsequently to study the differences between the two groups, the same analysis was carried out on the QCPR (training manikin's reports) results obtained immediately after the course as well as 6 months later.

For the qualitative assessment, the focus group was recorded and then transcribed. The textual corpus was analyzed by the researchers with the aim of highlighting the barriers and facilitators of the process.

### Dissemination and long term monitoring

In the following years, three additional schools gradually became involved in the initiative: the “Duillio Cambellotti” Institute in Rocca Priora (academic year 2018–2019), the Paolo Borsellino Institute in Montecompatri (academic year 2022–2023), and the Giovanni Pierluigi Institute in Palestrina (academic year 2022–2023). First, the heads of the schools were contacted by the research team, who explained the objectives and design of the study. Following the formal enrollment, BLSD courses (Table 1) were specifically organized for teachers. Following the model established during the pilot phase, selected teachers from each institution underwent comprehensive training to become educators (train-the-trainer model).

In all four schools, each educator received various pedagogical resources, including presentation slides, illustrated manuals, and instructional videos. To facilitate the teachers' work during the practical training, different supports were provided: Laerdal Little Anne QCPR manikins, which communicate via Bluetooth with the Laerdal QCPR app to provide instant feedback on the quality of chest compressions (depth, rate, and release); personal inflatable Mini Anne manikins for simultaneous training of students in a “mass training” mode; AED trainers. In every school, a project coordinator was designated and each institution was encouraged to maintain its involvement independently, with continuous monitoring to evaluate progress over time. INAIL researchers provided one retraining session per year for each teacher and were available to provide and update educational materials and devices.

Following a participatory research approach, the INAIL staff addressed the needs of the teachers and supported them by proposing or encouraging alternative solutions. The researchers attended as observers most of the courses delivered by the teachers, without interfering in classroom management. Several staff INAIL meetings were organized over the years to share information on the general progress of the project, the performance of the teachers, the management of educational activities (new courses and retraining). Much time was dedicated to updating the educational resources, especially during and after the COVID-19 pandemic. In the past 2 years, schools have also been asked to provide a written report of the activities carried out.

## Results

### Phases 1–4

The sample of participants trained is distributed as follows: 27 teachers at the BLSD course; eight of them attended the course for BLSD educators; 117 students (age 11–12), of which 56 trained by qualified instructors and 61 by trained teachers. The Mann-Whitney test calculated immediately after the courses revealed significant differences (*p* = 0.008) between the ability of the students trained by teachers and those trained by instructors after the course. The same comparison, 6 months later, led to different results (*p*-value Student's *T*-test = 0.40); the differences between the students trained by the teachers and those trained by INAIL instructors disappeared (Table 2). The same tests were conducted on QCPR results and they also showed significant differences between the group of teachers and that of the instructors, including total score, chest compressions and ventilations scores (Table 3). The same analyses were carried out 6 months after the conclusion of the course, but no significant differences emerged (Table 4). The analyses conducted between the two groups of students thus indicated that the initial differences diminished over time.

**Table 2:** Comparison between students trained by teachers vs. instructors through a hands-on skill test (immediately and 6 months after the course).

	**Teachers**	**Instructors**	***p*-value**
Skill test Mean (± SD)	20.85 ± 2.77	22 ± 2.58	Mann-Whitney *p* = 0.008^*^
Skill test after 6 months Mean (± SD)	15 ± 4.67	15.87 ± 5.27	Student's *T*-test *p* = 0.40

SD, standard deviation.

^*^Significant value.

**Table 3:** Comparison between students trained by teachers vs. instructors in the quality of chest compression and ventilations (after the course).

**After the BLS/AED course Mean (±SD)**	**Teachers**	**Instructors**	***p*-value**
Total score (%)	28.69 ± 18.92	38.23 ± 23.39	Student's *T*-test *p* = 0.017^*^
Chest compressions (%)	36.49 ± 34.92	55.54 ± 29.77	Mann-Whitney *p* = 0.004^*^
Ventilations (%)	53.29 ± 38.04	64.59 ± 36.67	Mann-Whitney *p* = 0.042^*^
Flow fraction (%)	59.95 ± 6.07	60.72 ± 7.28	Student's *T*-test *p* = 0.54

^*^Significant value.

**Table 4:** Comparison between students trained by teachers vs. instructors in the quality of chest compression and ventilations (after 6 months).

**After 6 months Mean (±SD)**	**Teachers**	**Instructors**	***p*-value**
Total score (%)	22.92 ± 21.91	25.22 ± 23.14	Mann-Whitney *p* = 0.58
Chest compressions (%)	32.69 ± 31.93	39.53 ± 33.77	Mann-Whitney *p* = 0.29
Ventilations (%)	37.39 ± 38.78	39.53 ± 39.66	Mann-Whitney *p* = 0.62
Flow fraction (%)	60.27 ± 18.07	61.64 ± 12.82	Student's *T*-test *p* = 0.67

During the focus groups, all the teachers expressed their willingness to continue the project independently, although some believed that an external expert might have piqued the students' curiosity more. The teachers also emphasized the need for a brief annual retraining to reinforce technical skills and self-confidence in transferring BLSD skills to students. More accustomed to proposing theoretical notions, the teachers found the hands-on approach useful. They did not encounter any difficulties in teaching the maneuvers and the training path was considered adequate to teach both the theoretical and practical skills. The didactic materials were deemed adequate and clear, but they requested additional resources, including videos relating to real cardiac arrest cases. Some teachers proposed reducing the hours of the BLSD course for organizational reasons. There was a general sense that the experience had positively engaged the children beyond expectations. Information circulated in the school, and 2nd-year students, excluded from the project for organizational reasons, insisting on being trained as well. Furthermore, thanks to contributions from local private sponsors, a short film entitled “We are all Jeeg Robot” was produced and participated in the international event “Catone Film Festival.”

### Phase 5 (long-term dissemination and monitoring)

Although significant differences were observed between the two groups immediately after the course, the decision was made to continue expanding the network of schools. The considerations are the following: the main themes that emerged from focus groups and field observations by INAIL instructors during the training sessions highlighted the teachers' strong motivation and aptitude for teaching BLSD. Finally, to involve more schools and ensure frequent retraining for already trained students, relying on teachers seemed the only feasible choice. This is because the INAIL staff could never guarantee such extensive coverage of students. To bridge the gap, teachers' technical knowledge and skills were reinforced through annual 1-h retraining sessions on practical maneuvers and by providing them with constantly updated didactic materials, including videos, annotated slides, and press reviews of successful sudden cardiac arrest interventions by bystanders.

New schools generally showed interest in joining and teachers were willing to participate in the training process, demonstrating that the topic is highly regarded in the school environment. Long- term observation have shown that appropriately trained educators can effectively advance the project: over 6 academic years (2017–2024), a total of 316 teachers were trained in BLSD (Basic Life Support and Defibrillation), 51 became educators, and 5,661 students were trained in life-saving maneuvers, with a ratio of one educator to 111 trained students. The only interruption in activities occurred during the COVID-19 pandemic.

All the teachers independently managed both theoretical lessons and hands-on training during BLSD courses. The coordinators proved to be essential for saving time and resources and motivating the other teachers. Each school also personalized the project based on their resources and organizational times. Teachers frequently requested updates on didactic materials and videos of cardiac arrest scenarios to show to the students. The equipment management is also an aspect to consider. The teachers appreciated the support provided by the research group in supplying them with mannequins and AED trainers that were always in order, charged and clean. Especially during practical training, new approaches were introduced that kept the students' attention high (Table 5): organizing CPR competitions using Laerdal QCPR mannequins paired with the app, introducing elements of peer education, organizing training and simulation stations during the school festival were among the solutions that found the greatest resonance and diffusion. The mass training method was also well-received as it allowed a larger number of students to be reached in less time. In some schools, the course was differentiated based on age: 1st-year students were taught using mass training, while 2nd and 3rd-year students had a 1–6 ratio. Overall, course durations were reduced in all schools: while in the pilot phase the courses lasted up to 5 h, in the regular project management, the time was reduced to no more than 2 h, even for courses conducted with a 1–6 ratio. Students responded enthusiastically to the teachers' proposals and, in some cases, even took on the challenge of teaching their younger peers.

**Table 5:** Innovative approaches to BLSD teaching—best practices from schools.

**Name**	**Description**	**Opportunities**
Peer to peer skill test	After practicing the maneuvers with the teacher's assistance, each student repeats the maneuver while peers complete the skill test and provide structured feedback.	Especially in the latter part of the lesson, when attention tends to wane, this technique keeps students engaged.
Peer to peer education	Third-year students were trained in BLSD maneuvers. The most capable students were selected to assist teachers during courses for 1st-year students.	Peer-to-peer education has shown significant benefits in other areas as well: it allows students to develop leadership, problem-solving, and team-building skills, and encourages the exchange of information and skills among students.
CPR competition	The Laerdal app, connected to multiple QCPR mannequins, allows for small team competitions. Each team is assigned a different-colored ambulance that advances on the screen based on the quality of the CPR performed on the QCPR mannequin. Two types of competitions were organized: - Relay race (faster): students line up and the instructor prompts a change every minute. - Scoring: each student performs CPR for 2 min against their peer from the other team. The team with the highest score wins.	Especially at the end of the lesson, this gamification technique consolidates acquired skills in a playful manner and keeps students' attention high until the end.
Training and awareness scenarios	During the school festival and community events, some students set up a scenario involving cardiac arrest (performing simulations with mannequins) and airway obstruction (having the unblocking maneuvers rehearsed on a mannequin). Bystanders can perform the maneuvers with the students' assistance.	This practice spreads BLSD culture within the community and enhances the students' sense of civic duty.
Mass training	Primarily for 1st-year students, CPR is introduced through mass training sessions. Each student is provided with a personalized inflatable mannequin and can perform the maneuvers as the teacher explains them, aided by slides and songs that match the correct chest compression rate.	This technique allows for significant time savings and a lower teacher-to-student ratio. Its effectiveness in terms of learning should be verified through comparison with standard methodologies.

The rotation of teachers, due to the precariousness of contracts or personal needs, risks having a negative impact. In the pilot school, for example, a critical moment occurred during the retirement and simultaneous transfer of the coordinator and another teacher to a different school. It was therefore necessary to train new teachers. In this case, the generational change had a positive impact: the new educators, all under 40, immediately got involved and are ensuring the sustainability of the project. In general, physical education and support teachers proved to be the most interested and motivated.

## Discussion

To enhance the population's ability to manage emergencies, life-saving techniques should be universally learned as automatic skills from a young age and teachers can play a crucial role. While the factors that hinder or facilitate teachers' engagement and motivation are well-covered in the literature (16, 22, 29), few qualitative studies have closely examined teachers' fieldwork following specific training (31). This study has highlighted critical learning points, organizational challenges, and adaptations over 6 years of field observation.

Many findings are consistent with previous research. Trained teachers serve as ideal multipliers of BLSD knowledge among students (9, 27): in our experience, for example, each teacher has trained 111 students. Head of schools and teachers generally perceive the topic as relevant, but their knowledge of BLSD is often scarce or absent (22, 28) and, especially in the initial phase, they may exhibit reluctance to assume responsibility (29). These barriers, however, have been overcome through field experience, particularly through the acquisition of practical skills.

In line with previous research (17, 27), our findings confirm that a few months after the training course, the skills students learned from teachers are not significantly different from those of students trained with qualified instructors. Some significant differences were observed immediately after the course; however, no other studies in the literature have conducted assessments at this stage of the process. The different result obtained at the two time points (immediately after the course and 6 months later) may be due to the gradual decline in skills over time, which led to a leveling of student performance, but further studies are needed. Additionally, the variability of situations and contexts could greatly influence the teaching abilities of both instructors (34) and teachers. In Germany, for example, the First Aid course is part of the teacher training curriculum, whereas in Italy it is not required by current regulations. Field observations revealed a steady improvement in the skills and BLSD abilities of teachers over time, which is particularly significant given that continuous retraining is a key component of the learning process (24).

Therefore, to ensure the project's sustainability, field observations have highlighted the need to: design structured pathways over time, enabling teachers to internalize the subject through hands-on training and regular retraining; carefully select teachers with a strong aptitude for practical teaching and a willingness to actively engage; provide continually updated and highly interactive didactic equipment; and identify internal coordinators, known as “early adopters” (29), who can motivate colleagues and coordinate activities.

These aspects have generated a positive climate. Once teachers gained basic confidence in their abilities, they overcame initial resistance and were able to design effective, engaging training courses for students, finding suitable solutions to organizational barriers. The students' interest, already highlighted in other studies (10), has proven to be a motivating factor for teachers, contributing to creating a dynamic and engaging atmosphere.

Therefore, while standardizing teacher training pathways is essential, our study shows that this is not required for students. Allowing schools to adapt paths to their organizational needs, once basic educational objectives are established, has proven to be a winning strategy, helping overcome barriers related to time constraints and curricular pressure. Schroeder et al. (10) also highlighted the role of teachers in adapting and improving the CPR training program over time. This flexibility, in perspective, could also favor the replication of the project in different socio-economic contexts. However, it is considered necessary to experiment with further forms of training to ensure that the acquired skills are maintained over time. The effectiveness of some techniques, such as mass training, which are widely used by teachers because they save time and human resources, remains to be evaluated.

## Limits

The observational nature of the study is both an advantage and a limitation. On one hand, it allows for in-depth exploration of internal organizational dynamics in schools, but on the other hand, it makes replicability more difficult: the relationship between researchers and teachers is one of mutual trust derived from the contingent circumstances. Moreover, the effectiveness of the training in the second phase of dissemination was no longer evaluated, especially considering some changes in the training models: reduction of time, use of mass training methods. The schools were selected within the same territorial area and thus share the same socio-demographic characteristics. Furthermore, INAIL provided schools with human and material resources free of charge; in other circumstances, they would need to raise the required funds.

## Conclusions

Teachers are ideal knowledge multipliers, but they need to be enabled to operate through continuous training, updated educational materials and adequate technological support.

Including BLSD skills in teachers' educational curricula could be useful, starting with some specializations. Our study highlighted, for example, a greater propensity of physical education and special needs teachers to get involved in this aspect, especially in hands-on training. However, one-time training, even if included in teachers' curricula, may not be sufficient, especially if it is only theoretical. To keep teachers updated and to share good practices, networks between the Ministry, the research community, training centers and schools should be implemented. In Italy, there are already courses for lay instructors promoted by associations operating nationwide through international guidelines and our study utilized one of these training courses. This provided a strong foundation for the methodology, with a view toward future standardization of a training pathway for teachers in Italy.

Future research perspectives should replicate the analysis of train-the-trainer processes in larger territorial areas to understand how such programs can be adapted and implemented in different socio-economic contexts and to identify the most effective strategies to overcome organizational and motivational barriers.

## Data Availability

The raw data supporting the conclusions of this article will be made available by the authors, without undue reservation.
